# Supplementary material for: Association between oral microbiome and seven types of cancers in East Asian population: a two-sample Mendelian randomization analysis
Source: Front Mol Biosci. 2023 Nov 21;10:1327893. doi: 10.3389/fmolb.2023.1327893 (PMC10702768; doi:10.3389/fmolb.2023.1327893)
Supplement: Supplementary file 1 [file DataSheet1.ZIP › Supplementary Materials/MR plots for tongue/tongue═╝/Pancreatic cancer/╥╚╧┘░⌐_╔╕╤í╜ß╣√/pheno.3612_to_pancreatic cancer_leave_one_out.pdf]

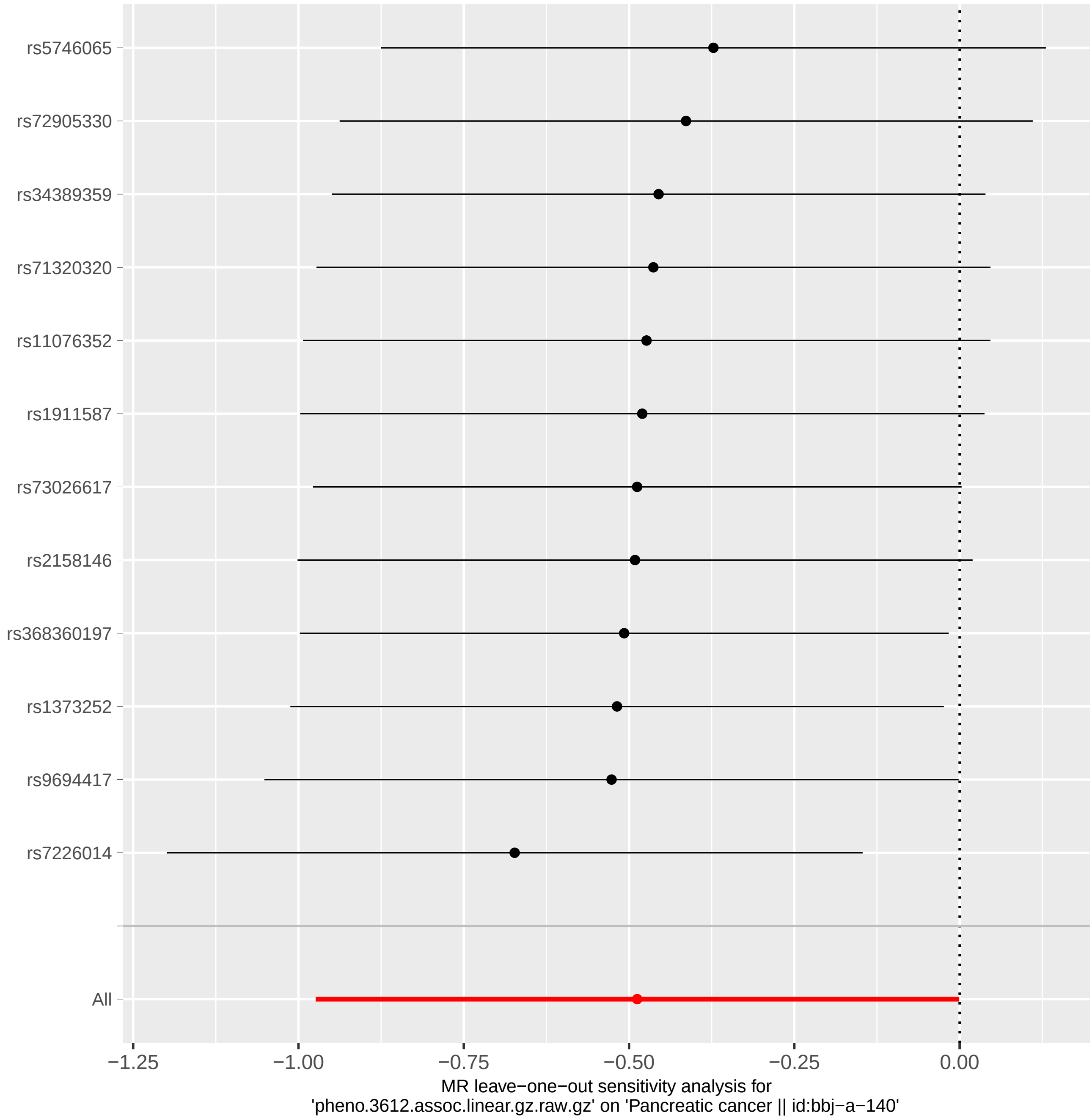
rs5746065
rs72905330
rs34389359
rs71320320
rs11076352
rs1911587
rs73026617
rs2158146
rs368360197
rs1373252
rs9694417
rs7226014
All
−1.25
−1.00
−0.75
−0.50
−0.25
0.00
MR leave−one−out sensitivity analysis for
'pheno.3612.assoc.linear.gz.raw.gz' on 'Pancreatic cancer || id:bbj−a−140'
